# In Vitro and Ex Vivo Synergistic Effect of Pyrvinium Pamoate Combined with Miltefosine and Paromomycin against *Leishmania*

**DOI:** 10.3390/tropicalmed9020030

**Published:** 2024-01-25

**Authors:** Estela Melcón-Fernández, Giulio Galli, Rafael Balaña-Fouce, Nerea García-Fernández, María Martínez-Valladares, Rosa M. Reguera, Carlos García-Estrada, Yolanda Pérez-Pertejo

**Affiliations:** 1 Departamento de Ciencias Biomédicas, Facultad de Veterinaria, Universidad de León, 24071 Leon, Spain; emelf@unileon.es (E.M.-F.); ggal@unileon.es (G.G.); rbalf@unileon.es (R.B.-F.); ngarf@unileon.es (N.G.-F.); rmregt@unileon.es (R.M.R.); cgare@unileon.es (C.G.-E.); 2 Instituto de Biomedicina (IBIOMED), Universidad de León, Campus de Vegazana s/n, 24071 Leon, Spain; 3 Instituto de Ganadería de Montaña (IGM), CSIC-Universidad de León, 24346 Grulleros, Spain; mmarva@unileon.es

**Keywords:** Leishmania, drug repurposing, drug combinations, pyrvinium pamoate plus miltefosine, pyrvinium pamoate plus paromomycin, intestinal organoids

## Abstract

One of the major drawbacks of current treatments for neglected tropical diseases is the low safety of the drugs used and the emergence of resistance. Leishmaniasis is a group of neglected diseases caused by protozoa of the trypanosomatidae family that lacks preventive vaccines and whose pharmacological treatments are scarce and unsafe. Combination therapy is a strategy that could solve the above-mentioned problems, due to the participation of several mechanisms of action and the reduction in the amount of drug necessary to obtain the therapeutic effect. In addition, this approach also increases the odds of finding an effective drug following the repurposing strategy. From the previous screening of two collections of repositioning drugs, we found that pyrvinium pamoate had a potent leishmanicidal effect. For this reason, we decided to combine it separately with two clinically used leishmanicidal drugs, miltefosine and paromomycin. These combinations were tested in axenic amastigotes of *Leishmania infantum* obtained from bone marrow cells and in intramacrophagic amastigotes obtained from primary cultures of splenic cells, both cell types coming from experimentally infected mice. Some of the combinations showed synergistic behavior, especially in the case of the combination of pyrvinium pamoate with paromomycin, and exhibited low cytotoxicity and good tolerability on intestinal murine organoids, which reveal the potential of these combinations for the treatment of leishmaniasis.

## 1. Introduction

Leishmaniasis is a group of neglected diseases caused by protozoan parasites of the *Leishmania* genus, which are widely spread in tropical and subtropical areas of the planet. Over twenty species of *Leishmania* have been described in different areas of the world that are transmitted by the bite of sandflies of the genera *Phebotomus* and *Lutztomya*, which act as vectors of the disease. The different species of these protozoa cause distinct clinical manifestations, which are grouped in three main types: visceral (VL), cutaneous (CL) and mucocutaneous leishmaniasis (MCL) [1]. During the life cycle, the parasite presents two main morphological stages: the promastigote form that is present in the sandfly vector, and the amastigote form, which develops inside the macrophages of the mammalian host [2]. 

The current treatment of leishmaniasis is based on chemotherapy, and the appearance of resistances has made the treatments used successfully in some places ineffective in others. The main treatments used against leishmaniasis are pentavalent antimonials (Sb^V^), amphotericin B (AMB), miltefosine (MTF) and paromomycin (PMM) [3]. Although Sb^V^ remains the first-line treatment for different types of leishmaniasis in many countries, it has significant drawbacks, such as the need for parenteral administration and the presence of serious side effects. Among those, the most worrying ones are cardiotoxicity and pancreatitis, and the loss of efficacy caused by the emergence of resistant strains long described in some geographical areas, such as the Indian state of Bihar [4,5]. AMB deoxycholate and the liposomal AMB formulation (AmBisome), are chemically unstable at the point of care, thereby requiring cold chain for delivery, and must be administered intravenously, due to their poor oral bioavailability [2,5]. However, they are very effective antileishmanial drugs and single large dose of AmBisome is recommended for VL treatment in the Indian subcontinent by the World Health Organization (WHO) after the loss of effectiveness of Sb^V^ [6]. Currently, there is a need to follow whether the single dose of the AmBisome regime can be associated with increased incidence of post-kala-azar dermal leishmaniasis (PKDL), a disfiguring sequel of VL [7]. MTF is the only oral drug approved for leishmaniasis, and has been used for VL, PKDL, CL, and HIV-VL coinfected patients [3,8]. Unfortunately, MTF has teratogenic issues, which prevents its use in pregnant women. In addition, its long half-life requires that women under this treatment must adopt contraceptive measures for at least 3 months after use [8]. Furthermore, there are studies showing the loss of MTF efficacy in India and Nepal after several years of use [9,10,11]. PMM is an aminoglycoside antibiotic used as a parenteral formulation for the treatment of VL, as well as in topical and parenteral formulations for the treatment of CL [8] but with limited efficacy as monotherapy [12]. However, PMM is effectively used in combination with Sb^V^, MTF and AmBisome [2,5]. In view of this situation, there is an urgent need to introduce new and more friendly drugs that meet the Target Product Profile where the disease is most prevalent [13].

The combination of drugs with different targets and synergistic effects, has been suggested as a strategy in the therapy for different pathologies. The synergistic effect of the drug combination can reduce the concentrations of each drug required to achieve the expected therapeutic effect, thus reducing the toxicity associated with each drug. Further, the different mechanisms of action provided by each drug involved in the combination reduce the generation of drug resistance processes [14]. The combination of Sb^V^ with PMM is recommended in East Africa for VL treatment [15,16]. Recently, the effectiveness of the combination MTF and PMM for VL treatment has been shown in this geographical area [15,17]. In the same way, combinations of AmBisome with MTF, AmBisome with PMM, and MTF with PMM are recommended for VL in the Indian subcontinent [2,5,18].

The combination of drugs increases the possibility of finding effective drugs using the repurposing strategy, since the reduction in the effective concentration makes it possible to reach the suitable plasma concentration [14]. Pyrvinium pamoate (PyP) is an FDA-approved drug for pinworm nematode infections with antiproliferative, antiparasitic and antimicrobial activities [19,20,21]. In a previous screening of two drug collections PyP was identified as a potent antileishmanial agent in an intracellular ex vivo platform [22]. The use of drug combinations with PyP could help overcome the problems associated with the oral bioavailability of PyP [23,24,25,26]. 

In the present work we have tested several combinations of MTF and PMM with PyP against free-living axenic amastigotes in vitro and intramacrophagic amastigotes living inside macrophages of ex vivo splenic explant, in order to find synergistic combinations between them capable of reducing their side effects and the generation of resistance. The cytotoxicity and tolerability of the combinations have been evaluated in mouse macrophages and in murine intestinal organoids, in order to assess their tolerability as potential oral drug.

## 2. Materials and Methods

### 2.1. Drugs

The standard antileishmanial drugs MTF (Sigma-Aldrich, Saint Louis, MO, USA) and PMM (Sigma-Aldrich, Saint Louis, MO, USA) were dissolved in sterile H_2_O at the concentrations of 12 mM and 100 mM, respectively. The antiparasitic agent PyP (Sigma-Aldrich, Saint Louis, MO, USA) was dissolved at a concentration of 10 mM in dimethyl sulfoxide (DMSO). All drugs were aliquoted and stored at −20 °C until use.

### 2.2. Parasites

For near-infrared detection of viable parasites, iRFP-*L. infantum*, a genetically modified strain of *L. infantum* BCN150 (MCAN/ES/96/BCN 150) that constitutively produces the bacteriophytochrome-based infrared fluorescent protein (iRFP) from *Rhodopseudomonas palustris*), was used throughout the experimental work. This strain was previously created in our laboratory [27,28]. The strain was maintained as free-living promastigotes in Schneider’s insect medium (Sigma-Aldrich, Saint Louis, MO, USA) supplemented with 20% (*v*/*v*) fetal bovine serum (FBS) (Gibco, Thermo Scientific, Waltham, MA, USA) and antibiotic cocktail (100 U/mL penicillin and 100 µg/mL streptomycin) (Hyclone, Thermo Scientific, Waltham, MA USA) at 26 °C, according to previous works [29,30], until it was used in mice infections.

### 2.3. Experimental Animals and Ethical Statement

In this work, Balb/c mice infected with iRFP-*L. infantum* were used to obtain axenic amastigotes from bone marrow and intramacrophagic splenic amastigotes. In addition, non-infected mice were used to obtain intestinal stem cells to initiate organoid cultures. The animals were acquired to Janvier Laboratories (St Berthevin Cedex, France) and maintained in the University of León animal house under standard housing conditions with free access to feed and water. The animal handling protocols used in this study complies with Spanish Act (RD 53/2013) inspired by European Union Legislation (2010/63/UE) and were approved by the Junta de Castilla y León under the authorization numbers OEBA 007–2019.

### 2.4. Experimental Infections and Set up of Primary Cultures

Six- to eight-weeks-old female Balb/c mice were inoculated intraperitoneally with 1.5 × 10^9^ infective iRFP-*L. infantum* metacyclic promastigotes. After 8 to 10 weeks postinfection, mice were sacrificed by cervical dislocation, and the femur and tibia of both legs as well as the spleen were aseptically dissected. 

The bones were cut at both ends and tempered phosphate buffered saline (PBS) was passed through the medullary cavity with a 29G syringe to extract the bone marrow cells [31,32]. The bone marrow cell suspension obtained was passed through a 100 µm-mesh cell strainer and centrifuged at 3500× *g* for 10 min at room temperature. In order to obtain the free amastigotes, cells were resuspended in amastigote medium containing 15 mM KCl; 136 mM KH_2_PO_4_; 10 mM K_2_HPO_4_ ·3H_2_O; 0.5 mM MgSO_4_ ·7H_2_O; 24 mM NaHCO_3_; 22 mM glucose; 1 mM glutamine, 1× Roswell Park Memorial Institute (RPMI) 1640 vitamin mix (Sigma-Aldrich, Saint Louis, MO, USA), 10 mM folic acid, 100 mM adenosine, 1× RPMI amino acid mix (Sigma-Aldrich, Saint Louis, MO, USA), 5 mg/mL hemin, antibiotic cocktail (100 U/mL penicillin and 100 µg/mL streptomycin) (Hyclone, Thermo Scientific, Waltham, MA, USA), 25 mM 2-Morpholinoethanesulphonic acid (MES), 10% FBS (Gibco, Thermo Scientific, Waltham, MA, USA), pH 5.6. Axenic amastigotes were routinely incubated at 36 °C.

Intramacrophagic amastigotes were obtained cutting spleens from experimentally infected Balb/c mice in small pieces and after incubation for 25 min with 5 mL of collagenase D (2 mg/mL) (Sigma-Aldrich, Saint Louis, MO, USA) prepared in buffer (10 mM 2-[4-(2-Hydroxyethyl)piperazin-1-yl]ethane-1-sulfonic acid (HEPES), pH 7.4, 150 mM NaCl, 5 mM KCl, 1 mM MgCl_2_ and 1.8 mM CaCl_2_). This suspension was passed through a 100 µm-mesh cell strainer, centrifuged at 500× *g* for 7 min at 4 °C, treated with lysis buffer (150 mM NH_4_Cl, 1 mM KHCO_3_, 0.1 mM EDTA, pH 7.1) and finally centrifuged three times at with a PBS wash between each centrifugation. Pellet was resuspended in RPMI medium (Gibco, Thermo Scientific, Waltham, MA, USA), supplemented with 20% FBS (Gibco, Thermo Scientific, Waltham, MA, USA), 1 mM sodium pyruvate, 24 mM NaHCO_3_, 2 mM L-glutamine, 1× RPMI vitamins (Sigma-Aldrich, Gillingham, UK), 25 mM HEPES and antibiotic cocktail [22].

### 2.5. Axenic and Intramacrophagic Amastigotes Viability Assays

To test the antileishmanial effect on axenic amastigotes, 1/2 or 1/3 serial dilutions of each drug or drug combination diluted in amastigote medium supplemented with 7.5% FBS (40 μL) were added to 384-well plates, each well containing 30,000 amastigotes in a volume of 40 μL. Thus, plates containing a volume of 80 µL per well were incubated at 36 °C and 5% CO_2_ during 72 h. 

In the case of intramacrophagic amastigotes, 40 μL of 1/2 or 2/3 serial dilutions of each drug or drug combination diluted in supplemented RPMI medium were added into 384-well testing plates, each well containing 40 μL of infected splenocytes. The plates were incubated at 36 °C and 5% CO_2_ for a maximum period of 144 h. 

A solution of 0.1% (*v*/*v*) DMSO and 10 μM AMB were used as negative and positive controls, respectively in each experiment. The viability of both parasite forms was assessed by means of the fluorescence emitted at 700 nm by the iRFP protein in living amastigotes, using an Odyssey (Li-Cor, Lincoln, NE, USA) infrared imaging system. It was assumed that 100% viability corresponds to the fluorescence emitted by negative control wells containing 0.1% (*v*/*v*) DMSO solution, whereas 0% viability corresponds to the fluorescence emitted by positive control wells containing 10 μM AMB.

EC_50_ values of each drug or drug combination were calculated plotting the fluorescence emitted by axenic or intramacrophagic amastigotes versus each concentration, using the nonlinear fitting analysis provided by SigmaPlot 10.1 statistical package [27].

### 2.6. Preparation of Murine Intestinal Organoids

Murine intestinal organoids cultures were prepared and maintained according to the protocols established by Stemcell Technologies™ (Vancouver, BC, Canada) (https://www.stemcell.com/intestinal-epithelial-organoid-culture-with-intesticult-organoid-growth-medium-mouse-lp.html#protocols; accessed on 22 January 2024) with some modifications. Briefly, a section of small intestine from humanely sacrificed animals was extracted and washed with ice-cold PBS to remove chyme or feces. Small slides (2 mm each) were cut, washed 15–20 times with ice-cold PBS and incubated with Gentle Cell Dissociation Reagent (Stemcell Technologies™, Vancouver, BC, Canada) for 20 min. After adding ice-cold PBS with 0.1% bovine serum albumin (BSA) to the slides and when they settle, the supernatant was filtered through a 70 µm strainer. This step was performed four times and the four fractions obtained were centrifuged at 300× *g* for 5 min at 4 °C and washed with ice-cold PBS + 0.1% BSA to collect the cells. After centrifugation, pellet was resuspended in 10 mL of ice-cold DMEM/F12 supplemented with HEPES 15 mM and glutamine (Gibco, Thermo Fisher Scientific, Waltham, MA, USA). A volume of 1 mL from each fraction was taken and analyzed under the microscope to select a clean fraction with the highest crypt number. The fraction was centrifuged at 200× *g* for 5 min at 4 °C and the pellet was resuspended in a 1:1 mix of Geltrex GFR LDEV-free (Gibco, Thermo Fisher Scientific, Waltham, MA, USA) and IntestiCult™ Organoid Grow Medium Mouse (Stemcell Technologies™, Vancouver, BC, Canada). 50 µL of this mix were pipetted into pre-heated 24-well plates, which were incubated at 37 °C for 10 min in order to promote the polymerization of the matrix. Finally, after adding 500 µL of IntestiCult™ Organoid Grow Medium Mouse, plates were incubated at 37 °C and 5% CO_2_. Organoids were split one into four plates after 7 days, and the culture medium was replaced every 48 h.

### 2.7. Cytotoxicity and Gut Tolerability of Drugs and Drug Combinations

To evaluate the cytotoxicity of the drugs or drug combinations, non-infected splenic cells and intestinal murine organoids were exposed to the highest concentrations of PyP, MTF and PMM, alone or in combination. 

A suspension of Balb/c mouse spleen cells obtained in the same way as indicated above, but derived from non-infected mice, was used to assess the cytotoxicity in splenic cells employing 96-well plates. Thus, 1 × 10^6^ cells were challenged with the highest concentrations of each drug or drug combinations prepared in RPMI medium. After 72 h of incubation at 37 °C with 5% CO_2_, cell viability was studied using the alamarBlue assay (Invitrogen, Thermo Scientific, Waltham, MA, USA).

Gut tolerability assays using murine intestinal organoids were made in 384-well plates, according to a protocol based on previous works [33]. The culture medium was discarded and 800 µL of Gentle Cell Dissociation Reagent (Stemcell Technologies™, Vancouver, BC, Canada) was added on top of matrix and incubated 1 min at RT. Then the matrix was disrupted and the suspension was incubated at RT for 10 min at 20 rpm. After that, this suspension was centrifugated at 300× *g* for 5 min at 4 °C. Discarding the supernatant, 10 mL of ice-cold DMEM/F12 (Gibco Thermo Fisher Scientific, Waltham, MA, USA) supplemented with glutamine and HEPES 15 mM were added and the suspension was centrifugated at 200× *g* for 5 min at 4 °C. Pellet was resuspended in a 1:1 mix of Geltrex GFR LDEV-free and IntestiCult™ Organoid Grow Medium Mouse (Stemcell Technologies™, Vancouver, BC, Canada) to obtain a 1:4 dilution in the final plate compared to the original culture which was seeded in a 384-well plate (8 µL/well) and incubated at 37 °C for 10 min. Then, 32 µL/well of IntestiCult™ Organoid Grow Medium Mouse (Stemcell Technologies™, Vancouver, BC, Canada) were added and the plate was incubated at 37 °C and 5% CO_2_. Mature organoids (4 days) were challenged with drugs, alone or in combination. Hydrogen peroxide (0.15% *v*/*v*) was used as positive control, whereas 0.2% DMSO was used as negative control. Viability of mini-guts after 72 h of exposure was determined by the AlamarBlue assay.

### 2.8. Statistical Analysis

The type of interaction derived from MTF/PyP and PMM/PyP when they are in binary combination was described by the median-effect/combination index (CI)-isobologram equation [33,34]. The CI < 1, = 1, and > 1 shows synergism, additive and antagonism effect of the combination, respectively. These interactions were analyzed with CalcuSyn software version 2.1. (Biosoft, Cambridge, UK).

## 3. Results

### 3.1. Effect of PyP Combined with MTF in Axenic Amastigotes

**Figure 1:**
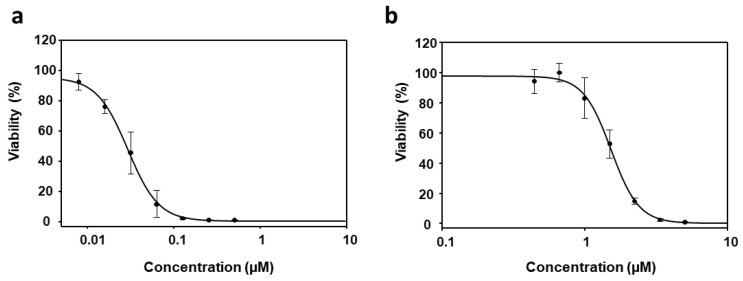
Dose–response curves of PyP (**a**) and MTF (**b**) tested in iRFP-*L. infantum* amastigotes obtained from bone marrow cells of infected mice. For PyP, 1/2 serial dilutions were prepared, starting from 0.5 µM. For MTF 2/3 serial dilutions were prepared, starting from 5 µM. Dose–response curves were adjusted with SigmaPlot software. Results show the mean values ± SD of three independent experiments with four technical replicates each.

To assess the in vitro effect of PyP combined with MTF, combination ratios were chosen based on the EC_50_ values obtained from individual PyP and MTF dose–response curves, which were determined measuring the infrared fluorescence emitted by axenic iRFP-*L. infantum* amastigotes exposed to these compounds (Figure 1a,b). The curves were fitted with the SigmaPlot software and showed EC_50_ values of 0.03 ± 0.00 µM for PyP and 1.53 ± 0.06 µM for MTF.

**Figure 2:**
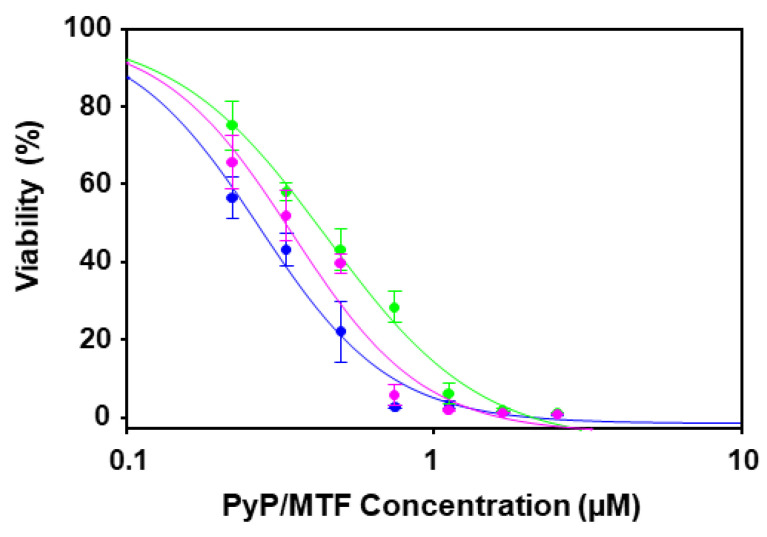
Dose–response curves of PyP/MTF 1/10 combination (blue line), PyP/MTF 1/20 combination (green line) and PyP/MTF 1/30 combination (pink line) tested in iRFP-*L. infantum* axenic amastigotes obtained from bone marrow cells. The starting concentrations in the PyP/MTF 1/10 combination were 0.25 µM/2.5 µM, with 2/3 serial dilutions. In the PyP/MTF 1/20 combination, the starting concentrations were 0.12 µM/2.5 µM, with 2/3 serial dilutions, whereas for the PyP/MTF 1/30 combination, the starting concentrations were 0.08 µM/2.5 µM, with 2/3 serial dilutions. Dose–response curves were adjusted with SigmaPlot software. Results show the mean values ± SD of three independent experiments with four technical replicates each.

Considering these results, the combination ratios assessed with axenic amastigotes extracted from bone marrow cells for PyP/MTF combination were 1/10, 1/20 and 1/30. The dose–response curves for these combinations are shown in Figure 2.

**Figure 3:**
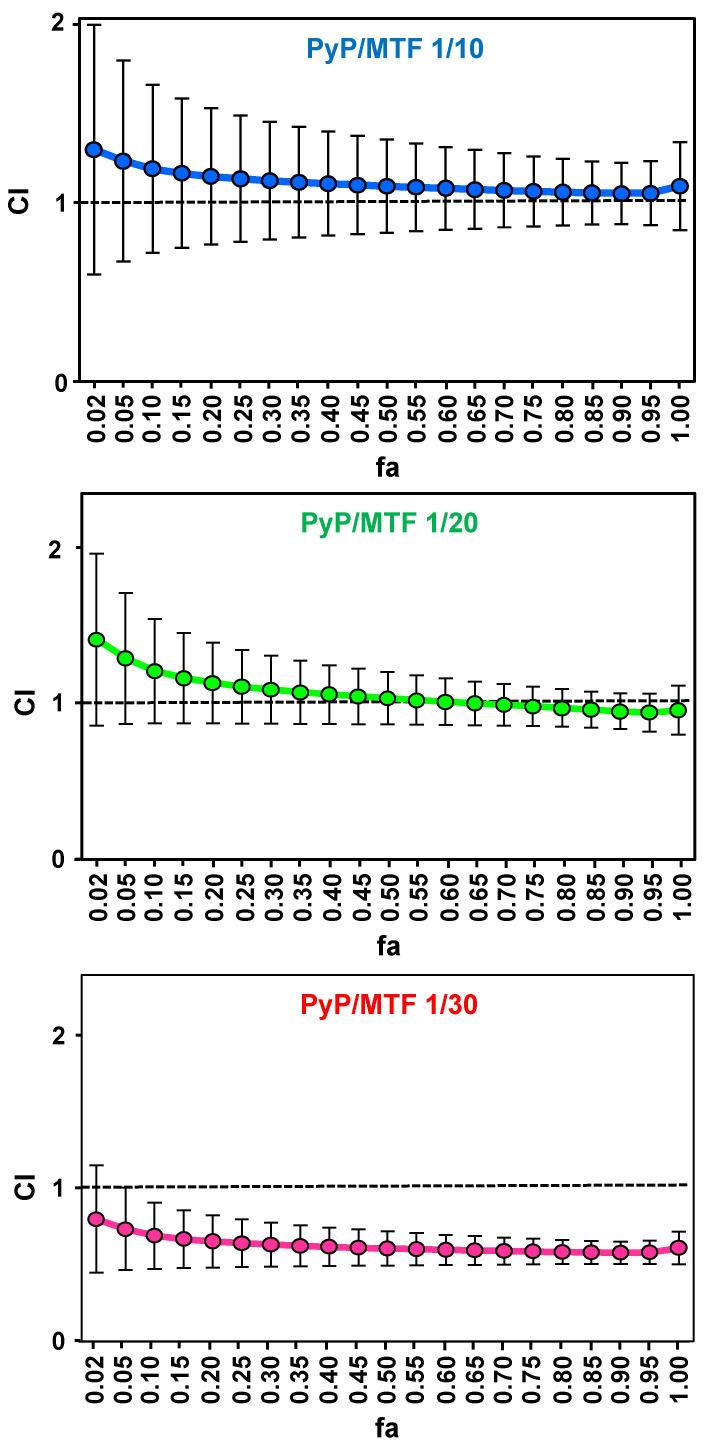
Interaction effect of three different combinations PyP/MTF (1/10, 1/20 and 1/30) in iRFP-*L. infantum* axenic amastigotes represented as CI (combination index) versus fa (fraction affected), obtained with the CalcuSyn software. The data show the mean of three different experiments with four technical replicates each.

The synergistic, additive or antagonist effect of the different combinations was analysed through the Chou-Talalay method and the CalcuSyn software [34,35], which provides “the combination index” (CI) to quantify the type of interaction (CI = 1: additive effect; CI < 1: synergism; and CI > 1: antagonism). This parameter is calculated from the dose–response curves adjusted with the software, which are defined by the parameters Dm (median-effect dose that inhibits the cells under study by 50%), m (coefficient signifying the shape of the dose–response curve) and r (correlation coefficient indicating the conformity of the data with the curve fitted). Figure 3 plots the CI values obtained for the different combinations versus the fraction affected (fa) (percentage of amastigote mortality) according to the Chou and Talalay method [34]. The CI values calculated for the 1/10 combinations were slightly higher than 1, hence having a nearly additive behavior, whereas the 1/20 combination showed slight synergism only at high concentrations (with fa values above 0.75), and the 1/30 combination resulted in a clear synergic effect (Figure 3, Table 1).

The dose-reduction index (DRI) measures how many times the dose of each drug can be reduced in the combination due to synergism [35]. Our results show that the PyP/MTF 1/30 combination allows at least a ~2-fold reduction in the PyP dose, and a ~3-fold reduction in the MTF dose at different effect levels of the dose–response curve (Table 2).

### 3.2. Effect of PyP Combined with MTF in Intramacrophagic Amastigotes

**Figure 4:**
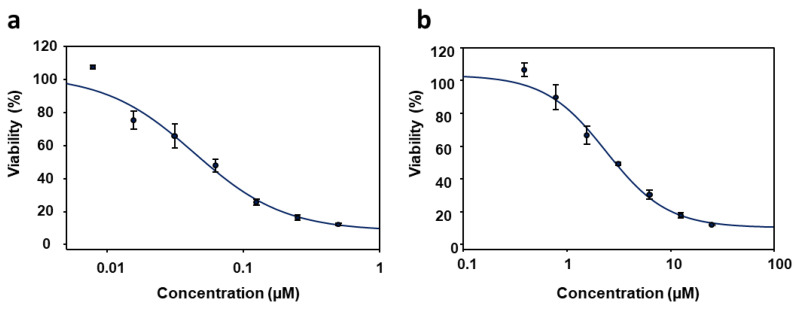
Dose–response curves of PyP (**a**) and MTF (**b**) tested in iRFP-*L. infantum* intramacrophagic amastigotes. For PyP, 1/2 serial dilutions were prepared, starting with 0.5 µM. For MTF 1/2 serial dilutions were prepared, starting with 200 µM. Dose–response curves were adjusted with SigmaPlot software. Results show the mean values ± SD of three independent experiments with four technical replicates each.

The PyP/MTF combinations that showed synergistic effects in axenic amastigotes (1/20 and 1/30) were tested in iRFP-*L. infantum* intramacrophagic amastigotes obtained from splenic explants of infected Balb/c mice. The antileishmanial activity of PyP and MTF alone in this new system was also calculated, and the EC_50_ values obtained from the dose–response curves (Figure 4a,b), which were fitted with the SigmaPlot software, were 0.04 ± 0.01 µM and 4.12 ± 0.25 µM, respectively.

**Figure 5:**
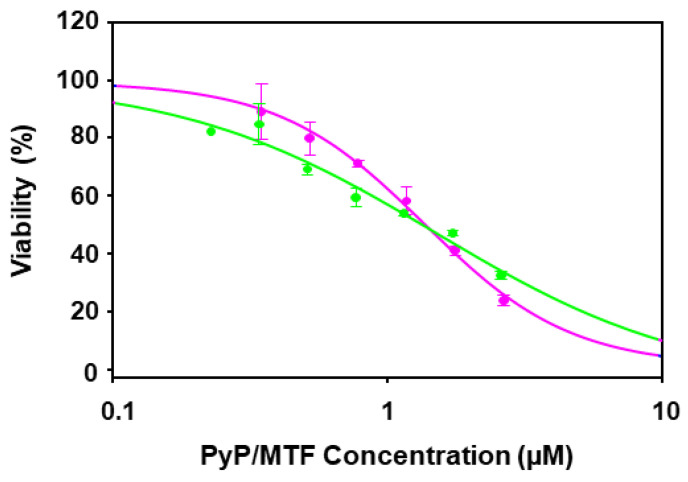
Dose–response curves of PyP/MTF 1/20 combination (green line) and PyP/MTF 1/30 combination (pink line) tested in iRFP-*L. infantum* intramacrophagic amastigotes. The starting concentrations in the PyP/MTF 1/20 combination were 0.12 µM/2.5 µM, with 2/3 serial dilutions, whereas for the PyP/MTF 1/30 combination, the starting concentrations were 0.08 µM/2.5 µM, with 2/3 serial dilutions. Dose–response curves were adjusted with SigmaPlot software. Results show the mean values ± SD of three independent experiments with four technical replicates each.

The dose–response curves corresponding to 1/20 and 1/30 PyP/MTF combinations tested against intramacrophagic amastigotes are shown in Figure 5.

**Figure 6:**
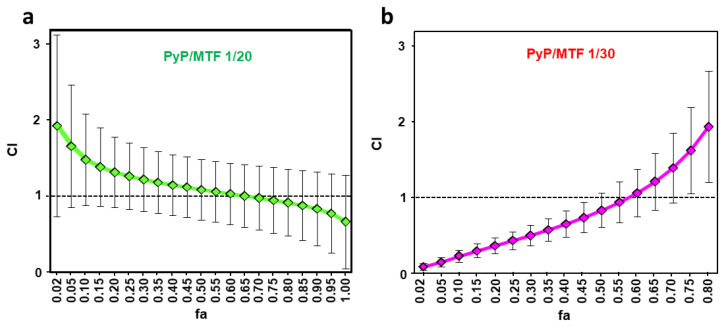
Interaction effect of two different combinations PyP/MTF 1/20 (**a**) and 1/30 (**b**) in iRFP-*L. infantum* intramacrophagic amastigotes represented as CI (combination index) versus fa (fraction affected), obtained with the CalcuSyn software. The data show the mean of three different experiments with four technical replicates each.

The synergistic, additive or antagonist effect of the different combinations was ana-lysed through the Chou-Talalay method and the CalcuSyn software, thus obtaining the CI values and the Dm, m and r parameters. Figure 6 plot the CI values obtained for the 1/20 and 1/30 combinations versus fa [34]. The CI values show that the 1/20 combination has a weak synergistic behavior only at higher concentrations, whereas the 1/30 combination is highly synergic at lower fa levels (Figure 6a,b, Table 3).

The DRI values calculated for the PyP/MTF 1/30 combination at the 25% of fa, indicated that this combination allows at least a ~3-fold reduction in the dose of both PyP and MTF (Table 4).

### 3.3. Cytotoxicity of PyP Combined with MTF in Spleen Cells and Intestinal Organoids

**Figure 7:**
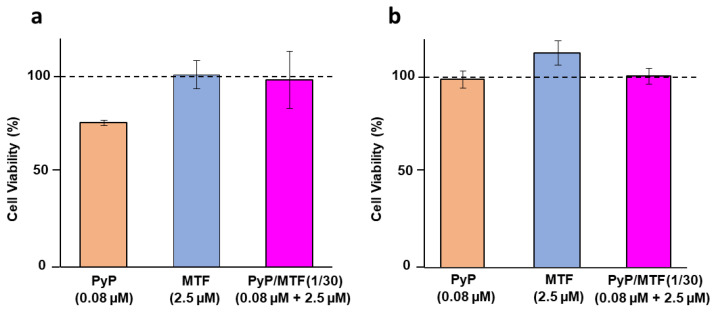
Cell viability of primary cultures of uninfected spleens (**a**) or intestinal murine organoids (**b**) in the presence of 0.08 μM PyP, 2.5 μM MTF and PyP/MTF (1/30 combination: 0.08 μM PyP + 2.5 μM MTF).

The cytotoxicity of combinations was assessed by measuring the viability of primary spleen cell cultures obtained from noninfected Balb/c mice, which were exposed to the highest concentrations of PyP and MTF present in the 1/30 combination. We observed that the viability of spleen cells was not compromised at the concentrations of drugs tested in combination, this viability been even higher than the viability of spleen cells exposed to PyP alone, although the statistical analysis did not show significant differences (Figure 7a).

Due to the possible oral administration of these two drugs in combination, the viability of murine intestinal organoids in the presence of the PyP/MTF combinations was tested. Figure 7b shows the viability of organoids at the highest concentrations of PyP and MTF present in the 1/30 combination. At these concentrations, organoid viability was not compromised and the toxicity of PyP alone was even lower than in spleen cells, showing the tolerability of this drug combination to be administered orally.

### 3.4. Effect of PyP Combined with PMM in Intramacrophagic Amastigotes

**Figure 8:**
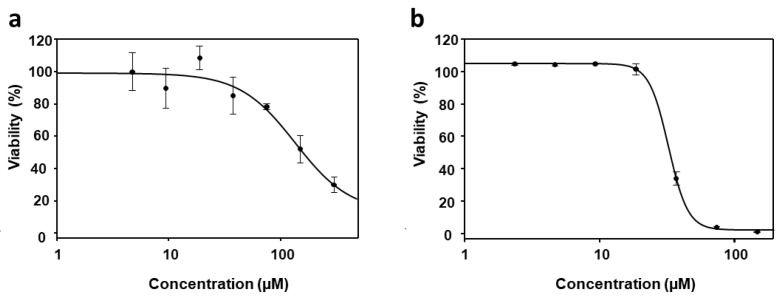
Dose–response curves of PMM tested either in iRFP-*L. infantum* amastigotes obtained from bone marrow cells of infected mice (**a**) or in intramacrophagic amastigotes (**b**). For axenic amastigotes, 1/2 serial dilutions were prepared, starting with 300 µM, whereas for intramacrophagic amastigores, 2/3 serial dilutions were prepared, starting with 125 µM. Dose–response curves were adjusted with SigmaPlot software. Results show the mean values ± SD of three independent experiments with four technical replicates each.

We also assess the efficacy of the PyP/PMM combination on both bone marrow axenic iRFP-*L. infantum* amastigotes and splenic explants infected with iRFP-*L. infantum*. Surprisingly, the effect of PMM on axenic amastigotes was extremely weak (EC_50_ > 100 µM), compared to the effect observed with intramacrophagic amastigotes obtained from primary mouse infected splenic cultures (EC_50_ = 28.10 ± 0.40 µM) (Figure 8a,b).

**Figure 9:**
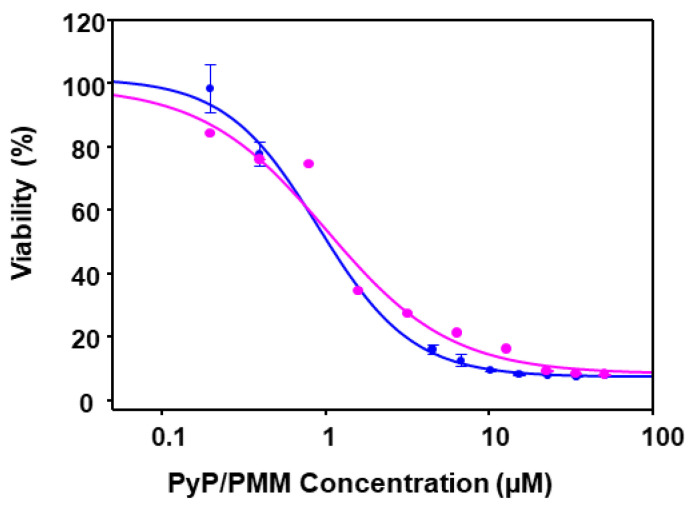
Dose–response curves of PyP/PMM 1/50 combination (blue line) and PyP/MTF 1/100 combination (pink) tested in iRFP-*L. infantum* intramacrophagic amastigotes. The starting concentrations in the PyP/PMM 1/50 combination were 1 µM/50 µM, with 2/3 serial dilutions, whereas for the PyP/PMM 1/100 combination, the starting concentrations were 0.50 µM/50 µM, with 2/3 serial dilutions. Dose–response curves were adjusted with SigmaPlot software. Results show the mean values ± SD of three independent experiments with four technical replicates each. The combinations tested were highly synergistic at almost any range of fa, showing CI values substantially < 1 (Figure 10a,b, Table 5).

Thus, combinations of PyP/PMM were assessed only in intramacrophagic amastigotes. Considering EC_50_ values for PyP and PMM, dose–response curves were prepared with data corresponding to 1/50 and 1/100 PyP/PMM combinations (Figure 9).

**Figure 10:**
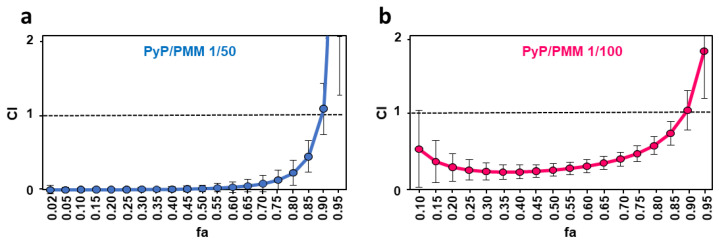
Interaction effect of two different combinations PyP/PMM 1/50 (**a**) and 1/100 (**b**) in iRFP-*L. infantum* intramacrophagic amastigotes represented as CI versus fa, obtained with the CalcuSyn software. The data show the mean of three different experiments with four technical replicates each.

**Table 5:** Efficacy values in intramacrophagic amastigotes and synergistic, antagonistic or additive effect of PyP, PMM and PyP/PMM combinations. Dm, m, r and CI parameters for the PyP/PMM combinations were obtained using the CalcuSyn software.

				CI Values at the Following Effect Levels			
Drug/s	Dm	m	r	25%	50%	75%	90%
PMM	8.96	0.36	0.96	*N/A	*N/A	*N/A	*N/A
PyP	0.02	0.34	0.88	*N/A	*N/A	*N/A	*N/A
PyP/PMM 1/50	0.02	0.34	0.88	0.003 ± 0.02	0.02 ± 0.05	0.13 ± 0.13	1.05 ± 0.33
PyP/PMM 1/100	0.51	0.57	0.98	0.25 ± 0.13	0.25 ± 0.08	0.46 ± 0.1	1.02 ± 0.25

*N/A: Not applicable.

In the case of PMM, the highest DRI values were obtained with the 1/50 combination, providing a score of at least near 300 in all the fa ranges, whereas for PyP, the highest DRI values were obtained in the 1/50 combination, with a score over 500 at 25% of fa (Table 6).

### 3.5. Cytotoxicity of PyP Combined with PMM in Spleen Cells and Intestinal Organoids

**Figure 11:**
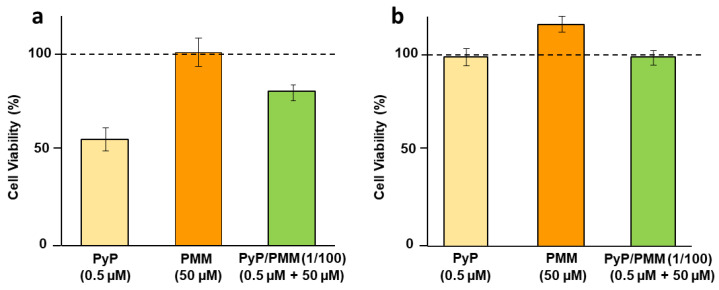
Cell viability of primary cultures of uninfected spleens (**a**) or intestinal murine organoids (**b**) in the presence of 0.50 μM PyP, 50 μM PMM and PyP/PMM (1/100 combination: 0.50 μM PyP + 50 μM PMM).

The viability of spleen cells in the presence of the highest concentrations of PyP and PMM in the 1/100 combination (Figure 11a) was only slightly affected, and reached 80% of the viability provided by the negative control. The concentration of PyP alone assayed in this combination was higher than that used in MTF combinations, therefore showing higher toxicity (about 50%) As in the case of the combination with MTF, the combination of PyP with PMM reduced its toxicity; however, the statistical analysis did not reveal significant differences (Figure 11a).

Regarding toxicity in mouse intestinal organoids, none of the concentrations tested were toxic, even when 0.5 μM PyP alone was used (Figure 11b).

## 4. Discussion

Drug resistance in VL treatment has been widely documented, one of the best examples being the loss of effectiveness of Sb^V^ in the Indian state of Bihar [18]. In fact, there are studies that indicate a failure rate of 65% in this region [36]. Although drug resistance is not the only factor responsible for treatment failure, there is substantial evidence that is a key issue and the administration for years of suboptimal doses of Sb^V^ has generated resistant parasites [37,38].

MTF and PMM are also susceptible to generating resistance, MTF being especially vulnerable due to is long half-life. Resistant strains could appear during this period of subtherapeutic concentrations in cases of relapsing patients or new infections. This drug was used in India, Nepal and Bangladesh as the basis of a VL elimination campaign, and after more than a decade of use, resistant cases and failure rates of 10% at 6 months and 20% at 12 months have been reported [39]. Regarding PMM, its use in monotherapy is not recommended, not only because of its low therapeutic efficacy when administered as monotherapy, but also to avoid the generation of resistances that are easily inducible in vitro [18,40]. 

Combination therapies have been used to minimize the generation of drug resistance. Thus, the use of two drugs with different mechanism of action can delay the process of drug resistance. In the case of VL treatment, this approach has overcome the therapeutic failure associated with Sb^V^ in the Indian Subcontinent, using combinations of liposomal AMB plus MTF, liposomal AMB plus PMM or PMM plus MTF [2]. If the two drugs have synergistic effects, generation of resistance is less likely, and the side effects associated with drugs is reduced, since their use in combination allows the decrease of the concentrations required to achieve therapeutic efficacy.

Currently, as mentioned above, therapies based on drug combinations have been included in the WHO recommendations for leishmaniasis treatment [2]. Also, this approach is currently used in Africa against late stages sleeping sickness by combining the ornithine decarboxylase inhibitor eflornithine [41] and the nitroheterocycle nifurtimox; NECT therapy [42]. Drug combination is not only a good strategy to reduce resistance, but also an interesting approach to increase the success of drug repurposing for neglected diseases. In this way, the union of drug repurposing and drug combination could be a useful strategy in the research of new, short, safe and cheap therapies for leishmaniasis [43].

PyP, an FDA-approved drug for pinworm infections, was identified as an antileishmanial agent after the screening of a library of compounds [22]. This compound also has activity against *M. tuberculosis* [20], *P. falciparum* [19], *C. parvum* [44], in addition to anticarcinogenic activity [26]. Initial studies carried out with pyrvinium and pamoate counterion as deworming showed similar therapeutic efficacy and less clinical toxicity than pyrvinium chloride [45]. 

The actual mechanism of action of PyP is not clear, but studies in *P. falciparum* have shown that the dissipation of the mitochondrial membrane potential caused by the drug would be at the origin of cell death [46,47]. Despite the potent leishmanicidal effect observed ex vivo, the in vivo action after its oral administration could be limited by the problems related to its bioavailability. There are uncertain data about the oral bioavailability of PyP. Early studies showed low or no concentrations of PyP in blood and urine after oral administration [23,24]. However, a more recent work in mice indicates, albeit with a high variability of results, that PyP would have a high volume of distribution and accumulate in fat and fatty tissues, with some systemic bioavailability when administered orally [26]. This would be in line with the efficacy of this compound demonstrated against several mouse models of cancer [48]. Therefore, the use of this compound in combination with another drug with synergistic effects, could be the solution to a putative problem of bioavailability, due to the reduction of the dose necessary to achieve the effect.

The two drugs currently used for the treatment leishmaniasis, which have been tested combined in this work, have pharmacological side effects. The main side effects associated with the use of MTF are nephrotoxicity, hepatotoxicity, gastrointestinal and teratogenic effects, whereas the use of PMM may cause nephrotoxicity, hepatotoxicity and ototoxicity [2]. The combination of PyP with MTF and PMM has shown synergistic effects, the latter being greater with PMM. In fact, all the combinations carried out with PMM were synergistic across almost the entire range of concentrations tested. This synergistic behavior, with DRI values higher than 1 for both MTF and PMM, would imply the reduction of PMM and MTF doses required to achieve the therapeutic effect. This is interesting, due to the need for new treatments safer than those currently used. Regarding PyP, some of the combinations also showed DRI values above 1. The reduction of the amount of PyP necessary to obtain the effect could reduce the problems associated with oral bioavailability of PyP. 

Further, the use of the combination of these drugs could reduce the risk of generation of resistance, because MTF, PMM and PyP act through different mechanisms of action, and in the three cases, involving multiple pathways. Thus, MTF inhibits lipid biosynthesis in parasites [49], affects mitochondrial cytochrome c oxidases [50], triggers an apoptosis-like cell death [51] and has effects in the TLR signaling of macrophages [52]. Regarding PMM, its mechanism of action has not been clearly identified; however, several studies have identified its interference with mitochondrial activity [53], as well as the inhibition of cytosolic protein translation due to its inhibitory and selective action on the leishmania ribosomes [54,55,56].

However, these are not the only mechanisms by which PMM exerts its action, and according to the results obtained in this study, they would involve the participation of some cellular pathway in the host, because the effect of PMM on axenic amastigotes was very weak compared to the observed with intramacrophagic amastigotes. This effect might be related to the fact that PMM can stimulate TLR4 to induce Th-1-biased immunomodulation. This has been observed in macrophages infected with *L. donovani* and treated with PMM, which releases TNF-α and nitric oxide in a TLR4-dependent manner [57]. As with MTF and PMM, the complete mechanism of PyP is unknown. The studies carried out with tumor cells, *P. falcuparum* or *C. parvum* indicate that PyP interferes with glucose uptake and mitochondrial activity by inhibiting NADH-fumarate reductase [58].

Observing the cytotoxicity values achieved from primary cultures of splenic cells, we found that the toxicity of PyP is reduced when tested in combination with MTF or PMM. We also note that the toxicity of PyP tested on intestinal organoids was lower than that shown against spleen cells. This could be a favorable data for its oral administration.

## 5. Conclusions

PyP has synergistic effects with MTF and PMM, this effect being higher with PMM. The use of this compound in combination therapies could reduce failure treatment associated with resistance generation and the side effects associated with current treatments based on MTF or PMM.

## Figures and Tables

**Table 1:** Efficacy values in axenic amastigotes and synergistic, antagonistic or additive effect of PyP, MTF and PyP/MTF combinations. Dm (median-effect dose), m (coefficient for shape of the dose–response curve), r (correlation coefficient) and CI (combination index) parameters for the PyP/MTF combinations were obtained using the CalcuSyn software.

Drug/s				CI Values at the Following Effect Levels			
	Dm	m	r	25%	50%	75%	90%
MTF	1.45	3.82	0.99	*N/A	*N/A	*N/A	*N/A
PyP	0.03	1.86	0.97	*N/A	*N/A	*N/A	*N/A
PyP/MTF 1/10	0.24	2.15	0.96	1.13 ± 0.35	1.09 ± 0.26	1.06 ± 0.35	1.05 ± 0.17
PyP/MTF 1/20	0.39	2.45	0.99	1.09 ± 0.23	1.02 ± 0.17	0.97 ± 0.13	0.94 ± 0.11
PyP/MTF 1/30	0.31	2.50	0.97	0.64 ± 0.16	0.60 ± 0.11	0.58 ± 0.08	0.58 ± 0.07

*N/A: Not applicable.

**Table 2:** Predictive dose-reduction index (DRI) calculated for the PyP/MTF combinations tested in amastigotes obtained from bone marrow cells, using the CalcuSyn software.

Drug Combination	DRI Values at the Following Effect Levels							
	25%		50%		75%		90%	
	MTF	PyP	MTF	PyP	MTF	PyP	MTF	PyP
PyP/MTF 1/10	7.51	1.00	6.00	1.08	4.79	1.17	3.83	1.27
PyP/MTF 1/20	4.34	1.16	3.69	1.33	3.14	1.54	2.68	1.77
PyP/MTF 1/30	5.48	2.19	4.71	2.55	4.05	2.97	3.48	3.46

**Table 3:** Efficacy values in intramacrophagic amastigotes and synergistic, antagonistic or additive effect of PyP, MTF and PyP/MTF combinations. Dm, m, r and CI parameters for the PyP/MTF combinations were obtained using the CalcuSyn software. Dm (median-effect dose), m (coefficient for shape of the dose–response curve), r (correlation coefficient) and CI (combination index) parameters for the PyP/MTF combinations were obtained using the CalcuSyn software.

Drug/s				CI Values at the Following Effect Levels			
	Dm	m	r	25%	50%	75%	90%
MTF	3.67	2.72	0.81	*N/A	*N/A	*N/A	*N/A
PyP	0.09	1.74	0.86	*N/A	*N/A	*N/A	*N/A
PyP/MTF 1/20	1.28	2.66	0.88	1.27 ± 0.44	1.09 ± 0.40	0.95 ± 0.43	0.84 ± 0.49
PyP/MTF 1/30	1.27	0.92	0.99	0.43 ± 0.11	0.83 ± 0.23	1.63 ± 0.57	3.23 ± 1.51

*N/A: Not applicable.

**Table 4:** Predictive dose-reduction index (DRI) calculated for the PyP/MTF combinations tested in intramacrophagic amastigotes, using the CalcuSyn software.

Drug Combination	DRI Values at the Following Effect Levels							
	25%		50%		75%		90%	
	MTF	PyP	MTF	PyP	MTF	PyP	MTF	PyP
PyP/MTF 1/20	2.90	1.09	2.87	1.35	2.84	1.67	2.82	2.08
PyP/MTF 1/30	6.43	3.61	2.90	2.04	1.31	1.15	0.59	0.65

**Table 6:** Predictive dose-reduction index (DRI) calculated for the PyP/PMM combinations tested in intramacrophagic amastigotes, using the CalcuSyn software.

Drug Combination	DRI Values at the Following Effect Levels							
	25%		50%		75%		90%	
	PMM	PyP	PMM	PyP	PMM	PyP	PMM	PyP
PyP/PMM 1/50	586.97	575.55	463.90	68.12	366.64	8.06	287.77	0.95
PyP/PMM 1/100	11.77	6.00	5.14	17.51	2.25	51.12	0.98	149.22

## Data Availability

Data is contained within this article.

## Abbreviations

VL: visceral leishmaniasis; CL: cutaneous leishmaniasis; MCL: mucocutaneous leishmaniasis; PKDL: post-kala-azar dermal leishmaniasis; Sb^V^: pentavalent antimonials; AMB: amphotericin B; MTF: miltefosine; PMM: paromomycin; PyP: pyrvinium pamoate.
